# Decapod Crustaceans in Animal Welfare Law: Fragmentation, Gaps, and Emerging Models in Europe and Oceania

**DOI:** 10.3390/ani16132061

**Published:** 2026-07-03

**Authors:** Lorenzo Fruscella, Diego Antonio Sicuso, Daria Vitale, Annamaria Passantino

**Affiliations:** 1 School of Design, University of Greenwich, Park Row, London SE10 9LS, UK; lorenzofruscella@yahoo.com; 2 Department of Veterinary Sciences, University of Messina, Via Palatucci Annunziata, 98168 Messina, Italy; diego150899@gmail.com; 3 Independent Researcher, Via Moltedo 72, 18100 Imperia, Italy; vitale.daria.segreteria@gmail.com

**Keywords:** Decapoda, invertebrates, sentience, animal welfare, animal protection, legal status, European Union, legislation

## Abstract

In recent decades, the exponential growth of the global seafood industry has intensified the exploitation of aquatic invertebrates, particularly decapod crustaceans, whose commercial value continues to rise alongside consumer demand. This expansion has coincided with significant scientific advances recognizing these animals as sentient beings capable of experiencing pain and exhibiting complex behavioral responses to noxious stimuli. Nevertheless, legal systems have been slow integrate such evidence, resulting in a persistent misalignment between scientific knowledge and regulatory practice. This article critically examines the legal status of decapod crustaceans within animal welfare law, underscoring a pronounced disjunction between well-established scientific evidence of their sentience and the limited protections they receive. Despite extensive research supporting their capacity for suffering, the prevailing legal paradigm continues to construe these animals predominantly as commodities. Through a comparative analysis of regulatory regimes across the European Union, Italy, and selected extra-European jurisdictions, including UK, Australia, and New Zealand, the study identifies a significant regulatory lacuna alongside considerable legislative fragmentation. It scrutinizes a spectrum of normative models, ranging from indeterminate, principle-based provisions to more exacting and technically prescriptive standards. The article finally advocates for a harmonized, science-driven regulatory framework to ensure coherent and effective welfare protections for decapod crustaceans within the global food industry.

## Simple Summary

The global consumption of decapod crustaceans is characterized by a paradox: while economic value and exploitation scales reach unprecedented levels, the legal protection afforded to these animals remains remarkably rudimentary. This regulatory inertia persists despite a robust body of scientific evidence identifying decapods as sentient beings capable of complex behavioral responses to noxious stimuli. This study provides a comparative analysis of the regulatory frameworks in the European Union, Italy, Austria, Switzerland, Norway, the United Kingdom, Australia, and New Zealand. We identify a significant regulatory gap where most jurisdictions still treat these animals as mere commodities. By evaluating different legislative models—ranging from vague principle-based norms to stringent technical mandates—this article argues for a harmonized, science-based approach to ensure effective welfare standards across the global food industry.

## 1. Introduction

The historical exclusion of decapod crustaceans from animal welfare legislation was traditionally predicated on the reductionist assumption that these invertebrates lacked the neurological complexity to experience pain. It was long contended that their reactions were limited to simple nociceptive reflexes—autonomous physiological responses to deleterious stimuli intended to facilitate escape, devoid of emotional or conscious processing. However, this anthropocentric paradigm has undergone a significant. As highlighted by Conte et al. (2021) [1], various species within the order Decapoda exhibit sophisticated behavioral responses to noxious stimuli that transcend reflexive action, providing compelling evidence of sentient perception. This shift was consolidated by the 2021 London School of Economics (LSE) report, commissioned by the UK Department for Environment, Food and Rural Affairs (DEFRA) [2]. After analyzing over 300 scientific studies, the report concluded that decapod crustaceans and cephalopod mollusks are sentient beings. The report defines sentience as the capacity to have feelings, including pain, pleasure, hunger, thirst, warmth, joy, comfort, and excitement.

Following this evidence, the British Veterinary Association (BVA) formally recognized decapod sentience, advocating for the mandatory use of “humane” slaughter methods. This evolving scientific consensus culminated in the United Kingdom’s Animal Welfare (Sentience) Act of 2022 [3], which legally enshrines the status of decapods as sentient beings.

The legislative developments in the United Kingdom are reflective of a broader, albeit inconsistent, international movement toward the recognition of invertebrate welfare. As early as 2005, the European Food Safety Authority (EFSA) issued a seminal report concluding that decapod crustaceans possess clear biological indicators of awareness. This includes the capacity for complex behaviors and the ability to experience pain, suffering, and distress. Consequently, the EFSA recommended that these animals be granted a level of protection against pain and suffering equivalent to that afforded to vertebrates [4].

The relevance of decapod crustacean welfare is further underscored by the scale of global exploitation, which involves tens of billions of individuals annually. Precise estimates remain difficult to establish, as industrial reporting is typically based on biomass rather than individual counts; nevertheless, available data indicate that decapods constitute one of the most extensively exploited groups of non-domesticated animals worldwide. This includes both wild-harvested species such as the European lobster (*Homarus gammarus*), the American lobster (*Homarus americanus*), and the brown crab (*Cancer pagurus*), as well as intensively farmed species, most prominently the white leg shrimp (*Penaeus vannamei*).

Across these production systems, welfare concerns arise at multiple stages of the production chain. In aquaculture, decapods are commonly subjected to high stocking densities, suboptimal water quality, and invasive reproductive interventions, including eyestalk ablation to induce spawning. In wild capture fisheries, individuals frequently experience prolonged air exposure, overcrowded storage on ice, physical injuries during trapping and handling, and pre-slaughter procedures such as tendon severing (“nicking”). Slaughter methods often involve live boiling or gradual freezing without prior stunning, practices increasingly considered likely to cause significant pain and distress in light of emerging scientific evidence.

Despite this growing body of evidence, legal protection remains highly inconsistent across jurisdictions, resulting in substantial regulatory gaps. While countries such as Switzerland, Austria, Norway, New Zealand, and several Australian states have introduced pioneering measures aimed at improving decapod welfare, the global crustacean industry—projected to reach a market value of USD 25.3 billion by 2031—continues to operate largely within an incomplete and uneven regulatory framework.

This persistent discrepancy between scientific knowledge and legal regulation highlights the need for a systematic reassessment of existing animal welfare regimes. It is within this context that the present study situates both wild-harvested and farmed decapod populations, as current legal frameworks play a decisive role in determining the welfare outcomes of billions of individuals.

The article aims to critically examine the legal status of decapod crustaceans through a comparative international lens, focusing on regulatory fragmentation across the European Union, Italy, Austria, Switzerland, Norway, the United Kingdom, Australia, and New Zealand. Specifically, the study seeks to: (*i*) identify and analyze the regulatory gaps at both supranational and national levels that allow for the continued exclusion of decapods from basic welfare protections; (*ii*) evaluate the effectiveness of different legislative approaches, distinguishing between broad welfare principles and detailed, science-based regulatory standards; and (*iii*) propose a harmonized regulatory framework for the European Union, drawing on “best practices” developed in leading jurisdictions, particularly Switzerland and New Zealand.

By integrating legal analysis with contemporary ethological and welfare science evidence, this study aims to contribute to the development of a coherent regulatory framework capable of facilitating a transition from a predominant commodity-oriented approach to one grounded in the recognition of sentience, ensuring that the treatment of decapod crustaceans aligns with current scientific understanding and evolving ethical standards.

## 2. The European Union: A Paradox of Advanced Protection and Systematic Exclusion

The European Union is currently undergoing a comprehensive revision of its animal welfare legislation under the framework of the Farm to Fork Strategy [5]. This ongoing overhaul represents a critical regulatory window in which long-standing inconsistencies within the existing legal architecture may be addressed. Supported by recent EFSA scientific opinions (https://www.efsa.europa.eu/en/topics/topic/animal-welfare?utm_source=chatgpt.com accessed on 7 April 2026) and historical European Citizens’ Initiatives [6], EU policymakers are increasingly under pressure to ensure that secondary legislation coherently reflects the sentience mandate enshrined in Article 13 of the Treaty on the Functioning of the European Union (TFEU) [7]. In this context, the potential expansion of the legal definition of “animal” beyond vertebrates within forthcoming legislative revisions could provide a historic opportunity to bridge persistent regulatory gaps and introduce harmonized, science-based baseline protections—such as technical standards on transport densities and pre-slaughter electronarcosis—across all Member States, thereby contributing to the emergence of a more unified regulatory standard. Despite this high-level constitutional recognition of animal sentience, a significant regulatory gap persists within EU secondary legislation. While Article 13 TFEU explicitly recognizes animals as sentient beings and requires full regard to their welfare requirements in policy formulation and implementation, its practical translation remains largely confined to vertebrate species. As a result, decapod crustaceans continue to occupy a position of legal invisibility within the EU framework, effectively remaining in a regulatory limbo.

This exclusion is not merely an oversight but is codified across the core pillars of EU animal welfare law. For instance, Council Directive 98/58/EC [8], which establishes general minimum standards for the protection of animals kept for farming purposes, explicitly excludes “any invertebrate” from its scope (Art. 1, par. 2, let. d). This categorical exclusion creates a structural inconsistency in which the principle of sentience is formally acknowledged at the Treaty level, yet simultaneously denied operational effect for taxa that scientific evidence increasingly indicates are capable of experiencing pain and distress.

The regulatory fragmentation is further compounded within the legal frameworks governing transport and slaughter. Council Regulation (EC) No 1/2005 [9] on the protection of animals during transport applies exclusively to “live vertebrate animals”, thereby excluding decapod crustaceans from any harmonized welfare safeguards during often extensive international supply chains characterized by high density confinement, thermal stress, and prolonged sensory deprivation.

Similarly, Council Regulation (EC) No 1099/2009 [10] on the protection of animals at the time of killing ignores invertebrates entirely, thereby permitting slaughter methods such as live boiling or gradual freezing prior stunning.

By treating decapod crustaceans primarily as agricultural products under Annex I of the TFEU, without corresponding recognition of their biological and behavioural characteristics, the EU has produced a fragmented regulatory landscape. In the absence of a unified directive, Member States have developed divergent national approaches, resulting in legal inconsistency that undermines both the coherence of the, internal market and the ethical aspirations underpinning contemporary animal welfare policy. Against this backdrop, the ongoing revision of the EU animal welfare legislation: constitutes a pivotal moment: it offers the possibility of reconciling the sentience mandate of Article 13 TFEU, with regulatory practice, thereby integrating decapod crustacean into a coherent, science-based supranational protection framework.

## 3. National Legal Frameworks: A Comparative Analysis of Fragmentation

The international legal landscape regarding decapod crustaceans is characterized by extreme heterogeneity, reflecting a transitional phase between the traditional “commodity-based” view and an emerging “sentience-based” paradigm. In the absence of harmonized international standards, national legislations vary from total exclusion to sophisticated, science-based technical regulations.

### 3.1. Italy: Regulatory Fragmentation and the Role of the Judiciary

To analyze the national framework, it is first necessary to define regulatory fragmentation. In this context, it refers to intra-national, localized inconsistency where distinct municipal or regional entities within a single, unified national legal system enact disjointed or contradictory local rules, causing commercial uncertainty.

The Italian legal system exemplifies the challenges associated with a decentralized and incomplete approach to the regulation of decapod crustacean welfare. At present, Italy lacks a comprehensive national statute specifically addressing the protection of decapod crustaceans, resulting in a regulatory vacuum at the primary legislative level. This absence has led to the emergence of a fragmented “patchwork” of municipal regulations (“Regolamenti comunali”), generating significant legal uncertainty for operators in the food production and food service sectors. In practice, while certain urban jurisdictions have introduced prohibitions concerning the maintenance of live lobsters on ice or their immersion in boiling water without prior stunning, adjacent municipalities may not impose any equivalent restrictions, thereby producing a highly inconsistent regulatory landscape. Within this context, it remains standard commercial practice to store live decapods—most notably the European lobsters (*Homarus gammarus*) and the American lobsters (*Homarus americanus*)—in conditions of immobilization on ice, often with mechanical restraint of the chelae, followed by slaughter through live immersion in boiling water. Although such practices are not explicitly prohibited under national primary legislation, their legality has been increasingly questioned under Article 544-ter Italian Penal Code, which sanctions the maltreatment of animals in cases involving “unnecessary suffering” or conditions deemed “incompatible with the biological characteristics of the species.”

A harmonized and updated EU animal welfare regulation would serve as the structural resolution to this domestic fragmentation. Given that EU Regulations are directly applicable and do not require national transposition, a uniform supranational framework would automatically prevail over inconsistent municipal provisions and fragmented local ordinances. This would effectively eliminate the current “patchwork” effect, providing legal certainty for operators in the food industry and restaurant sector, ensuring a level playing field across jurisdictions, and establishing clear and enforceable compliance criteria throughout the entire national territory.

Within the Italian context, such supranational harmonization would be particularly significant, as it would resolve the existing regulatory ambiguity between local administrative initiatives and general criminal law provisions. It would also strengthen legal coherence by aligning domestic enforcement practices with EU-level animal welfare standards, thereby reducing interpretative divergence among local authorities and judicial bodies.

#### 3.1.1. Municipal Regulations and the “Patchwork” Effect

The governance of decapod welfare in Italy is currently delegated to local authorities. Under the authority granted by the Testo Unico delle Leggi sull’Ordinamento degli Enti Locali (Legislative Decree 267/2000) [11] and underpinned by constitutional regulatory powers, Italian municipalities have the autonomy to enact animal welfare ordinances. However, the legal efficacy of these provisions is strictly confined to their respective municipal territories, resulting in a geographically inconsistent legal landscape.

A comprehensive review by Liuzzo et al. (2017) [12] examined 110 Italian administrative centers to evaluate the prevalence of decapod-specific protections. Of the 62 animal welfare regulations identified, 46 contained provisions applicable to aquatic animals and crustaceans. The study revealed a heterogeneous regulatory environment: while some municipalities provide exhaustive specifications regarding tank dimensions, water oxygenation, and salinity, others focus on slaughter methods, such as the explicit prohibition of live boiling.

**Figure 1:**
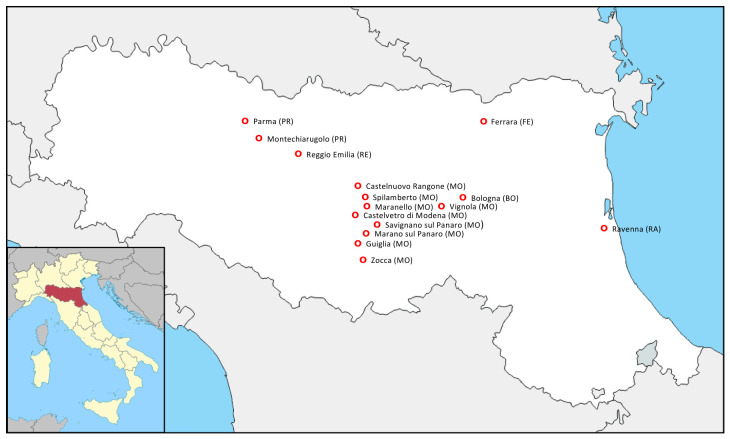
Municipalities of the Emilia-Romagna region in Italy where regulations are in force for the protection of decapod crustaceans, with municipal codes in parentheses.

The region of Emilia-Romagna exemplifies this regulatory disharmony. Within this single regional territory, neighboring municipalities—often separated by only a few kilometers—enforce vastly different and occasionally contradictory standards (Figure 1).

For instance, while the Municipality of Montechiarugolo (2021) [13] adopts advanced technical guidelines derived from regional Lombardy ordinances (D.G.R. X/6196) [14], other nearby centers like Ferrara, Parma, and Reggio Emilia maintain distinct sets of criteria for transport and storage.

This “patchwork” distribution of norms generates significant legal uncertainty for food operators and hampers effective enforcement. The current framework is characterized by a reliance on diversified scientific references of varying degrees of modernization, failing to offer a uniform standard of protection.

#### 3.1.2. The Role of the Judiciary: Case Law as a Regulatory Substitute

In the absence of a unified national statute, the Italian judiciary has increasingly functioned as a regulatory substitute through the strict interpretation of criminal law. A prominent example is the landmark precedent set by the Italian Court of Cassation (Cass. Pen., Section III, n. 27173/2016) [15], which upheld a conviction under Article 544-ter of the Penal Code against a food service operator for keeping live lobsters on ice.

Drawing heavily on scientific findings from the National Reference Centre for Animal Welfare (IZSLER), the Court determined that near-zero degree storage is fundamentally incompatible with the biological nature of the species and induces unjustifiable suffering [16].

This jurisprudential trend was further reinforced in 2019 near Milan, where a restaurateur found storing a live lobster on ice was sentenced to three months of socially useful work (probation) [17]. These rulings represent a critical shift, identifying a clear mandate for the minimization of suffering. However, relying on case-by-case judicial intervention remains an inefficient substitute for comprehensive, primary legislation.

#### 3.1.3. Constitutional Mandate for Reform

The necessity for a unified national intervention is further emphasized by the recent amendment to Article 9 of the Italian Constitution, formally approved in 2022 [18], which explicitly elevates environmental and animal protection to the rank of fundamental constitutional principles. The newly introduced provision explicitly mandates that the State shall regulate the “modes and forms” of animal protection, thereby introducing a clear constitutional duty to develop coherent and scientifically grounded regulatory frameworks. This constitutional shift suggests that reliance on fragmented municipal ordinances is no longer sufficient to ensure consistent and effective protection. To align with modern ethological evidence and constitutional imperatives, Italy requires a cohesive national framework that transcends municipal boundaries and provides science-based welfare protection for decapod crustaceans throughout the production and distribution chain.

By explicitly recognizing that animals are no longer to be regarded merely as economic utilities or objects of human property, the constitutional amendment repositions them as beings entitled to intrinsic protection. This normative reconfiguration represents a substantive departure from a strictly utilitarian legal paradigm and establishes a more direct connection between constitutional values and animal welfare regulation. Importantly, this domestic constitutional evolution aligns closely with the aforesaid principles enshrined in Article 13 TFEU, thereby strengthening the coherence between national and supranational legal orders. In doing so, it provides the Italian legal system with a reinforced constitutional basis to effectively integrate and implement forthcoming EU-wide animal welfare reforms, ensuring smoother regulatory convergence and reducing interpretative friction between national and European legal frameworks.

### 3.2. Australia: The Impact of Jurisdictional Divergence

In contrast to localized fragmentation, the Australian framework is characterized by jurisdictional divergence. This term defines a structural federated model where legally independent state and territory legislative bodies deliberately exercise their sovereign rights to adopt entirely different statutory definitions of what constitutes an “animal”. The regulatory framework for animal welfare in Australia is characterized by a significant degree of jurisdictional decentralization. While the Australian Constitution does not grant the Commonwealth government an explicit, direct head of power to legislate for animal welfare, the federal government can utilize indirect constitutional mechanisms to enact welfare standards. Specifically, the Commonwealth can leverage its trading corporations power and external affairs power to regulate animal welfare, as observed in federal live export regulations. Furthermore, under Section 109 of the Australian Constitution, validly enacted Commonwealth laws automatically override conflicting state legislation, though day-to-day animal welfare enforcement continues to be largely delegated to individual state and territory statutes.

The legal protection of decapod crustaceans in Australia is fundamentally contingent upon the statutory definition of “animal” adopted within each jurisdiction. This creates a binary landscape of protection.

Exclusionary jurisdictions: such as South Australia, Queensland, and Western Australia adopted a restrictive definition of “animal” limited to vertebrate species, thereby excluding crustaceans from basic welfare protections. Conversely, inclusionary jurisdictions—including the Australian Capital Territory (ACT), New South Wales (NSW), Victoria, and the Northern Territory—extend legal recognition to crustaceans, albeit often subject to specific statutory conditions or regulatory limitations (Table 1).

Among these, Victoria and the Northern Territory adopt the most comprehensive approach, encompassing all decapod crustaceans (and, in the Northern Territory, all crustaceans) within their protective scope. By contrast, Queensland, New South Wales, and the ACT rely on detailed classificatory frameworks to determine which categories of crustaceans fall within legislative oversight.

Where inclusion applies, animal welfare statutes generally, prohibit acts of cruelty and the infliction of “unreasonable and unnecessary pain”, often supplemented by non-exhaustive lists of prohibited conduct, including beating, maiming, torturing, or exposing animals to excessive thermal stress.

In New South Wales, Victoria, and the ACT, commercial operators are additionally subject to statutory “duty of care” obligations requiring the provision of adequate food, water, and shelter. During transport, operators must implement measures to prevent injury and physiological distress, while sector-specific requirements imposed on retailers and restaurateurs include: (*i*) the maintaining hygienic storage environments to prevent disease and injury; (*ii*) prohibiting the sale of severely diseased or injured shellfish; and (*iii*) avoiding rapid fluctuations in water temperature and separating incompatible species to minimize stress [19,20,21].

An analysis of Australia’s legislative landscape reveals, however, a stark geographical paradox between regions of intensive decapod production and those offering the most developed welfare protections. Major shrimp and prawn aquaculture hubs are heavily concentrated in northern and subtropical regions, most notably Queensland. Yet Queensland’s regulatory framework remains largely centred on generic industry codes of conduct and does not provide the same level of statutory animal welfare protection afforded in more inclusionary jurisdictions. By contrast, jurisdictions such as NSW and the ACT, which implement more advanced welfare legislation and duty of care obligations, function primarily as major consumer and retail markets rather than principal production areas. This reveals a structural disconnect between the geographical loci of intensive decapod rearing and those jurisdictions in which the most robust legal protections are enforced.

The practical application of these laws reached a significant milestone in 2017 with the conviction of Nicholas Seafood (RSPCA v Seafood-Buyfood Pty Ltd. Trading as Nicholas Seafood Traders), a prominent business in Sydney, NSW. This case represented the first known instance of a successful prosecution for animal cruelty involving crustaceans in Australia. The proceedings originated from an inspection conducted by the Royal Society for the Prevention of Cruelty to Animals (RSPCA NSW), prompted by video evidence of a worker butchering a spiny lobster while it was still conscious and without prior stunning, in breach of the Prevention of Cruelty to Animals Act (NSW). Although the defendant initially contested the penalty, the court upheld the conviction for animal cruelty and imposed a penalty of 1500 Australian dollars. Following the verdict, the company implemented mandatory staff training to ensure “humane” killing methods in alignment with government welfare guidelines [27]. This case underscores that, even within jurisdictions possessing relatively advanced statutory frameworks, effective protection of decapod crustaceans, remains dependent on judicial enforcement and regulatory oversight to bridge the gap between formal legal standards and industrial practice.

### 3.3. General Welfare Principles: Norway and Austria

Other European nations have adopted a more explicitly “principle-based” approach to decapod crustacean welfare. In Norway, the Animal Welfare Act explicitly states that its provisions apply to “crustaceans,” acknowledging their intrinsic value regardless of their utility to humans [28]. This legislative choice reflects a gradual shift toward a more biocentric legal philosophy, in which welfare protection is grounded in the sentient status of animals rather than their economic function. Similarly, Austria includes decapods crustacean within its general welfare obligations, focusing on the prevention of “unjustified pain or suffering” without, however, developing an extensive set of species-specific technical standards.

The distinct legislative trajectories observed in Norway and Austria are closely linked to their respective socio-economic structures and industrial profiles. Norway is a major global exporter of marine resources, including large quantities of deep-water prawns and red king crabs. In this context, the early inclusion of crustaceans within the Norwegian Animal Welfare Act can be interpreted as serving a dual regulatory objective: on the one hand, ensuring compliance with high domestic ethical standards, and on the other, reinforcing the international competitiveness of Norwegian seafood exports in markets increasingly sensitive to sustainability and welfare credentials. By contrast, Austria, as a landlocked state with negligible domestic decapod production and relatively low consumption levels, has developed its inclusionary approach primarily in response to strong societal ethical norms and consumer-driven welfare expectations, rather than industrial or export-oriented pressures. This contrast illustrates that robust legal recognition of decapod welfare can emerge independently of local production intensity.

While these models represent a significant normative advancement compared to jurisdictions that continue to exclude invertebrates from their regulatory scope, they often remain limited in terms of technical operationalisation. In both cases, the legal framework establishes a general “duty of care” without consistently specifying the concrete biological and environmental parameters necessary for effective implementation in industrial contexts. Critical variables such as water oxygenation thresholds, temperature control during transport, stocking densities, or species-specific electrical stunning parameters are often left undefined, thereby creating interpretative uncertainty and enforcement variability in practice.

The divergence between principle-based recognition and technical regulatory precision highlights a broader challenge in the governance of decapod welfare: namely, the difficulty of translating ethical recognition into enforceable, science-based standards capable of ensuring consistent protection across production, transport, and slaughter systems.

### 3.4. The United Kingdom: Enshrining Sentience into Statutory Law

The regulatory landscape in the United Kingdom experienced a profound paradigm shift following the publication of the London School of Economics (LSE) report in 2021. Commissioned by the Department for Environment, Food and Rural Affairs (DEFRA), the report provided robust neuroscientific and behavioral evidence confirming that decapod crustaceans and cephalopod mollusks possess the capacity to experience pain, distress, and pleasure.

This scientific consensus led directly to the enactment of the Animal Welfare (Sentience) Act 2022, which legally expanded the definition of “animal” to include all decapod crustaceans within its scope. Unlike principle-based models that treat animals primarily under generic welfare obligations, the UK framework formally mandates that policy-making bodies must consider the adverse effects of prospective regulations on the welfare of these newly recognized sentient beings.

However, similar to the challenges observed in other transitioning jurisdictions, a gap still remains between the high-level constitutional recognition of sentience under the 2022 Act and the implementation of specific, legally binding technical mandates governing daily commercial transport, storage on ice, and mandatory pre-slaughter electronarcosis in the gastronomic sector.

## 4. Advanced Regulatory Models: Switzerland and New Zealand

While most jurisdictions limit their legal oversight to broad, non-binding declarations of intent, Switzerland and New Zealand have achieved a significant paradigmatic shift by translating the recognition of sentience into rigorous, enforceable operational protocols. These two models represent the “vanguard” of decapod protection, moving beyond vague welfare principles to adopt specific technical mandates grounded in measurable scientific evidence.

The prominence of Switzerland and New Zealand within the international regulatory landscape can be attributed not only to their recognition of decapod sentience, but also to their ability to operationalize that recognition through coherent and adaptable legal frameworks. In both jurisdictions, animal welfare regulation is informed by a precautionary approach that prioritizes the prevention of suffering even where scientific uncertainty persists. Rather than relying solely on broad ethical declarations, legislators have progressively incorporated scientific findings into enforceable standards governing transport, husbandry, handling, and slaughter practices.

This regulatory philosophy stands in contrast to many decentralized systems, where welfare protections are often fragmented across jurisdictions or remain confined to general duties of care.

### 4.1. The Swiss Technical and Professional Model

The Swiss legislative framework represents one of the most advanced global examples of decapod welfare protection, transitioning from general principles to specific, enforceable technical mandates.

#### 4.1.1. Legislative Framework: AniWA and OPAn

On 16 December 2005, the Swiss Federal Assembly approved the Animal Welfare Act (AniWA) [29] While Article 2 initially limits its scope to vertebrates, it grants the Federal Council the discretionary power to extend protections to specific invertebrates, predicated on contemporary scientific knowledge regarding the sentience of invertebrate animals. In accordance with this mandate, the Federal Council issued the Ordinance on the Protection of Animals (OPAn) in 2008 [30]. Significant amendments were adopted in 2018 to bolster the respectful treatment of animals within the veterinary and gastronomic sectors. Notably, the scope of the OPAn currently encompasses decapod crustaceans of the clade Reptantia and the infraorder Procarididea, while specifically excluding the infraorders Stenopodidea and Caridea.

#### 4.1.2. Mandatory Welfare Standards and Prohibited Practices

Under the 2018 revisions [31], Switzerland effectively banned several traditional culinary practices. Live decapods protected under the ordinance can no longer be transported on ice or in ice slurry, nor can they be maintained in aquatic environments outside of water. Furthermore, the legislation imposes a strict stunning mandate prior to slaughter, which legally prohibits the immersion of conscious decapods in boiling water. General welfare principles under the OPAn also apply to these species, including mandates for adequate nutrition and care, the mandatory prevention of disease and injury, and requirements for social contact for species identified as social animals. Furthermore, breeding objectives must prioritize health and the absence of characteristics detrimental to the animal’s dignity.

#### 4.1.3. Professional Requirements and Technical Specifications

The Swiss model emphasizes professional competence by stipulating that the professional keeping and breeding of decapods may only be performed by personnel who have attained specialist training. This ensures that operators possess the technical knowledge and practical skills necessary for respectful treatment. Transport containers must maintain species-specific water quality, and handling must be minimized to ensure animals remain in an aquatic or sufficiently moist environment during sorting. Crucially, Article 177 mandates that decapods be killed only by expert personnel. Stunning is compulsory, with electronarcosis and the mechanical destruction of the brain identified as the primary approved methods. The Federal Food Safety and Veterinary Office (FSVO) further clarifies that because of the specialized knowledge required for humane slaughter, the sale of live decapods to private individuals is no longer appropriate; instead, animals should be sold frozen or killed immediately by trained professionals [32].

#### 4.1.4. Evaluation of FSVO Technical Guidelines

The FSVO provides rigorous technical guidelines that codify permissible and prohibited actions. Approved killing methods are limited to electrocution, electronarcosis followed by boiling, or electronarcosis followed by the mechanical destruction of the nerve centers. Conversely, the mechanical destruction of nerve centers without prior stunning is prohibited, as is the sectioning of live animals, the use of microwave or steam cooking on conscious subjects, and various forms of suffocation via air, carbon dioxide, or non-aerated water. The guidelines also explicitly forbid freezing as a primary killing method, the placement of marine decapods in freshwater (or vice versa), and the use of cold storage as a substitute for stunning. For specific species like crabs, the FSVO notes that mechanical destruction is anatomically demanding and carries a high risk of delayed death, therefore failing to meet welfare requirements [33].

While the Swiss system exhibits regulatory gaps—most notably the exclusion of certain shrimp infraorders—it provides an unprecedented level of protection. By integrating scientific evidence of sentience into clear technical prohibitions, Switzerland has established a regulatory benchmark that aligns commercial gastronomic practices with modern ethical and biological realities. The Swiss legal system thus guarantees a superior level of animal protection compared to most international jurisdictions, primarily through the modification of the OPAn in 2018 which introduced stringent rules ensuring decapod crustaceans enjoy protections once reserved only for vertebrates.

### 4.2. The Dynamic Evolutionary Model of New Zealand

New Zealand’s regulatory system, governed by the Animal Welfare Act 1999 and subsequent 2018 amendments [34,35], is characterized by its remarkable capacity to evolve in response to emerging scientific literature. Protection extends not only to crabs and lobsters but also to kōura (freshwater crayfish), reflecting a broader ecological sensitivity toward endemic species. The New Zealand model emphasizes humanitarian slaughter standards by requiring that animals be rendered unconscious and maintained in that state until death. A defining feature of this system is its dynamic nature; for instance, in 2021 [36] the authorities officially removed slow freezing from the list of acceptable humane stunning methods following neuroscientific evidence that it failed to induce immediate anesthesia and instead caused prolonged suffering through tissue damage. Beyond the point of slaughter, the model incorporates strict monitoring of environmental parameters, such as water quality and oxygenation levels during commercial storage.

Ultimately, the New Zealand framework underscores the importance of the precautionary principle, dictating that where scientific uncertainty exists regarding the degree of suffering, the law must intervene in favor of the maximum possible protection for the animal.

In summary, both Switzerland and New Zealand demonstrate that the protection of decapod crustaceans is not merely an ethical imperative but a concrete regulatory possibility. These models provide a robust blueprint for future harmonization within the European Union, proving that transitioning to more humane handling and slaughter methods does not compromise industry viability but rather elevates its ethical and qualitative standards.

To provide a structured synthesis of these variations and identify specific regulatory benchmarks, Table 2 maps the integration of decapod protections against key technical and legal indicators across all examined jurisdictions.

## 5. Discussion and Policy Recommendations

The comparative analysis conducted in this study reveals a profound discrepancy between the scientific consensus on decapod sentience and the current state of global animal welfare legislation.

While the sentience shift is biologically undeniable, the legal response remains a fragmented patchwork of insufficient protections. This regulatory inertia not only perpetuates practices that cause significant physiological and psychological distress but also creates legal uncertainty for international trade and the gastronomic industry.

**Figure 2:**
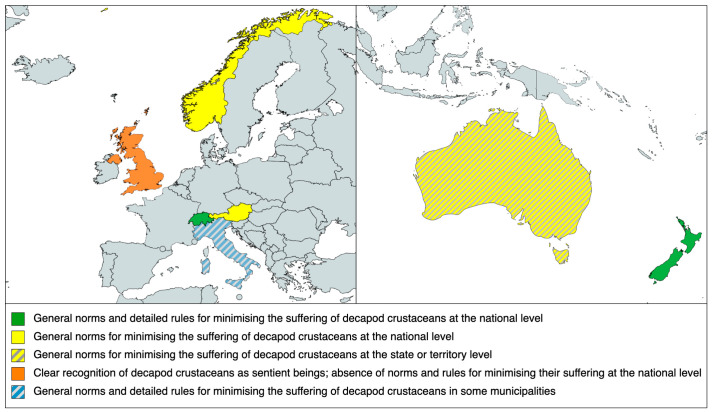
The different degrees of protection offered to decapod crustaceans within the legal frameworks analysed in Europe and Oceania.

This regulatory inertia not only perpetuates practices that cause significant physiological and psychological distress but also creates legal uncertainty for international trade and the gastronomic industry. To provide a comprehensive geographical overview of these disparities, Figure 2 illustrates the varying degrees of protection identified across the examined zones in Europe and Oceania.

To bridge the gap between ethological evidence and legislative practice, this study proposes an integrated regulatory model based on four synergistic pillars. First, there must be an explicit Legal Recognition of Sentience; as demonstrated by the United Kingdom’s recent reforms, formally including decapod crustaceans within the definition of “animal” is a necessary prerequisite for any further protection. Second, the adoption of the Intrinsic Value Principle, as seen in Norway, is essential to shift the legal paradigm from treating crustaceans as mere economic commodities to recognizing them as individual beings with their own welfare requirements.

However, formal recognition remains symbolic without Technical Standardization. The models provided by Switzerland and New Zealand prove that it is both possible and practical to implement measurable parameters for water quality, oxygenation, and temperature during transport and storage. Finally, the most urgent intervention required is the Mandatory Implementation of Humane Slaughter. Prohibiting live boiling and slow freezing in favor of electrical stunning or rapid mechanical destruction is the only way to align industrial practices with the precautionary principle.

Given that this study intentionally focuses on the world’s primary importing and consuming blocks (such as the European Union) alongside legislative vanguards (Switzerland, New Zealand, Australia), the regulation of import activities represents a powerful trade lever for global change. Major shrimp- and decapod-exporting nations often lag in domestic welfare laws. However, by introducing strict border welfare controls and incorporating animal welfare clauses into international trade and bilateral agreements, importing jurisdictions can effectively compel global production markets to elevate their operational standards, leveraging economic market access to protect sentient life worldwide.

Crucially, the regulatory fragmentation and structural gaps identified within the global food industry extend their negative impacts into scientific research environments. In many of the examined jurisdictions, such as New South Wales, animal research statutes explicitly restrict the definition of an ‘animal’ to vertebrates, leaving decapod crustaceans entirely outside the scope of institutional ethical oversight. This creates a severe ethical paradox: a decapod is recognized as a sentient being capable of suffering when handled in a commercial kitchen or retail market, yet the exact same organism is stripped of legal protection and ethical monitoring when subjected to invasive laboratory procedures, underscoring the urgent need for a unified, cross-sector legal definition of animal sentience.

For the European Union, the current revision of animal welfare legislation represents a historic opportunity to lead this global transition. While EU institutions are already engaged in this overhaul, this study directly enhances the ongoing legislative process by providing a rigorous, comparative roadmap for implementation. Formally recognizing sentience under Article 13 of the TFEU remains a symbolic gesture unless secondary legislation is reinforced with specific, technically measurable parameters—such as explicit bans on slow-freezing and mandatory pre-slaughter electronarcosis—modeled after the successful frameworks of Switzerland and New Zealand.

Furthermore, by mapping the commercial uncertainty caused by Italy’s localized fragmentation, this analysis provides empirical justification for the adoption of directly applicable EU Regulations rather than Directives, ensuring a level playing field across member states. Ultimately, by addressing critical regulatory blind spots such as international trade clauses for importing countries and ethical oversight within scientific research laboratories, this review offers policymakers the necessary framework to ensure that the updated EU legislation does not merely react to political pressure, but structurally reflects modern biological realities and ethical mandates. The transition to more humane standards does not merely serve an ethical mandate; it enhances the integrity and sustainability of the entire food production chain.

## 6. Conclusions

This study critically evaluated the legal status of decapod crustaceans by contrasting modern ethological evidence of sentience with current regulatory structures in Europe and Oceania. Our comparative analysis answered the core research objectives by successfully mapping the regulatory gaps in supranational frameworks like the EU, detailing the challenges of local regulatory fragmentation in Italy and jurisdictional divergence in Australia, and evaluating the technical mandates implemented by vanguard nations like Switzerland and New Zealand.

In conclusion, resolving the widespread exclusion of these animals requires a definitive shift from commodity-based frameworks to science-driven, harmonized legal protection models. Implementing centralized, technically prescriptive standards remains the only viable path to ensure that the global food and retail industry operates in alignment with modern ethical imperatives and biological realities.

## Figures and Tables

**Table 1:** Legislative framework and specific rules for crustaceans in Italy and major Australian states and territories.

Country/State/Territory	Act (Year)	General Rules	Definition of ‘Animal’ Includes Crustaceans (Yes/No)	Definition of ‘Animal’ Concerning Crustaceans
Italy (National & Municipal Framework)	Legislative Decree 267/2000; Penal Code Art. 533-ter	There is a total absence of a unified national statute. Welfare governance is heavily fragmented and delegated to local authorities through municipal regulations (Regolamenti comunali), resulting in a geographical patchwork of protections.	No (At national statutory level)/Yes (At fragmented local municipality levels)	Legally excluded from national welfare statutes. However, local ordinances in specific municipalities and penal case law explicitly target practices like live boiling or maintaining live lobsters on ice, treating them as biological maltreatment
State of New South Wales	Prevention of Cruelty to Animals Act (1979) [19]	It prohibits cruelty to animals and covers vertebrate animals and in particular cases crustaceans. The act allows for the adoption of additional rules for different species of animals.	Yes	Includes crustaceans, but ‘*only when at a building or place (such as a restaurant) where food is prepared or offered for consumption by retail sale in the building or place*’.
Australian Capital Territory	Animal Welfare Act (1992) [20]	It prohibits cruelty to animals, and applies to vertebrate animals, cephalopods, and crustaceans.	Yes	Includes live crustaceans intended for human consumption.
State of Victoria	Prevention of Cruelty to Animals Act (1986) [21]	Forbids cruelty to all vertebrates and some decapod crustaceans.	Yes	Includes lobsters, crabs, and crayfish.
State of Queensland	Animal Care and Protection Act (2001) [22]	Prohibits acts of cruelty, and establishes a duty of care applied to vertebrates, specific cephalopods, and crustaceans belonging to the class Malacostaca.	Yes	Includes members of the class Malacostraca. Crabs, lobsters, crayfish, and prawns are given as examples.
State of South Australia	Animal Welfare Act (1985) [23]	It prohibits harming animals ‘*intentionally, unreasonably or recklessly*’. The act applies to vertebrate animals only, with the exception of fish.	No	Includes only vertebrates, with the exception of humans and fish.
Territory of Northern Australia	Animal Protection Act (2018) [24]	It prohibits causing unnecessary suffering to animals, and establishes a duty of care. The act does not specifically address the welfare of farmed animals.	Yes	Includes all crustaceans.
State of Western Australia	Animal Welfare Act (2002) [25]	Forbids unnecessary acts of cruelty and harm to animals, including abandonment. The law covers vertebrates, excluding fish.	No	Includes live invertebrates ‘*of a prescribed kind*’, which is however not specified.
State of Tasmania	Animal Welfare Act (1993) [26]	Forbids taking any action that it may cause unreasonable and needless pain or suffering to an animal. This applies to all vertebrate animals. The law offers rules on the transport and slaughter of several species.	No	Includes any living vertebrate other than a human beings, or any other creature specified for the purposes or provisions of the act, under which, however, crustaceans are not mentioned.

**Table 2:** Comparative synthesis of decapod crustacean legal protections and technical indicators across examined jurisdictions.

Jurisdiction	Legal Recognition of Sentience	Pre-Slaughter Stunning Mandate	Transport/Storage on Ice Prohibited	Main Regulatory Tool
European Union	Yes (Principle—TFEU Art. 13)	No (Excludes Invertebrates)	No	Secondary Legislation Gaps
Italy	No (Fragmented local level)	No (Only in certain Municipalities)	Fragmented (Regulated via Judiciary)	Local Ordinances & Penal Case Law
Australia	Divergent (State-by-State)	No (Except via business internal guidelines)	Divergent	State Welfare Acts (e.g., POCTA)
Norway	Yes (Intrinsic Value)	Yes	Indirectly via general care rules	Animal Welfare Act
Austria	Yes (General Principle)	No explicit technical mandate	No explicit rule	General Welfare Obligations
Switzerland	Yes (Centralized Mandate)	Yes (Mandatory)	Yes (Banned)	Animal Protection Ordinance (OPAn)
United Kingdom	Yes (Statutory Level)	No explicit nationwide technical mandate	No explicit rule	Animal Welfare (Sentience) Act 2022
New Zealand	Yes (Dynamic Model)	Yes (Mandatory)	Yes (Banned)	Animal Welfare Act & Care Regulations

## Data Availability

No new data were created or analyzed in this study. Data sharing is not applicable to this article.
